# Long-range enhancement of N501Y-endowed mouse infectivity of SARS-CoV-2 by the non-RBD mutations of Ins215KLRS and H655Y

**DOI:** 10.1186/s13062-022-00325-x

**Published:** 2022-06-05

**Authors:** Yichao Zhu, Wenzhao Zhou, Zubiao Niu, Jiayi Sun, Zhengrong Zhang, Qinqin Li, You Zheng, Chenxi Wang, Lihua Gao, Qiang Sun

**Affiliations:** 1 Laboratory of Cell Engineering, Research Unit of Cell Death Mechanism, Beijing Institute of Biotechnology, Chinese Academy of Medical Sciences (2021RU008), 20 Dongda Street, Beijing, 100071 China; 2 The Experimental High School Attached to Beijing Normal University, Beijing, China

**Keywords:** SARS-CoV-2, N501Y, H655Y, Ins215KLRS, Omicron, Cross-species transmission, Mouse infection

## Abstract

**Background:**

Rodents, such as mice, are vulnerable targets, and potential intermediate hosts, of SARS-CoV-2 variants of concern, including Alpha, Beta, Gamma, and Omicron. N501Y in the receptor-binding domain (RBD) of Spike protein is the key mutation dictating the mouse infectivity, on which the neighboring mutations within RBD have profound impacts. However, the impacts of mutations outside RBD on N501Y-mediated mouse infectivity remain to be explored.

**Results:**

Herein, we report that two non-RBD mutations derived from mouse-adapted strain, Ins215KLRS in the N-terminal domain (NTD) and H655Y in the subdomain linking S1 to S2, enhance mouse infectivity in the presence of N501Y mutation, either alone or together. This is associated with increased interaction of Spike with mouse ACE2 and mutations-induced local conformation changes in Spike protein. Mechanistically, the H655Y mutation disrupts interaction with N657, resulting in a less tight loop that wraps the furin-cleavage finger; and the insertion of 215KLRS in NTD increases its intramolecular interaction with a peptide chain that interfaced with the RBD-proximal region of the neighboring protomer, leading to a more flexible RBD that facilitates receptor binding. Moreover, the Omicron Spike that contains Ins214EPE and H655Y mutations confer mouse infectivity > 50 times over the N501Y mutant, which could be effectively suppressed by mutating them back to wild type.

**Conclusions:**

Collectively, our study sheds light on the cooperation between distant Spike mutations in promoting virus infectivity, which may undermine the high infectiousness of Omicron variants towards mice.

**Supplementary information:**

The online version contains supplementary material available at 10.1186/s13062-022-00325-x.

## Background

According to the World Health Organization (WHO), as of April 1, 2022, coronavirus disease 2019 (COVID-19), caused by the novel severe acute respiratory syndrome coronavirus 2 (SARS-CoV-2), had infected more than 486 million patients, posing a major threat to global public health and safety (https://covid19.who.int). Part of patients with COVID-19 developed severe conditions that are closely related to cellular catastrophe and aberrant immune [1–3]. Clinical manifestations include fever, cough, anosmia, pneumonia, cytokine storm, expansion of myeloid-derived suppressor cells, lymphopenia and so on [4–6].

The SARS-CoV-2 genomic RNA encodes for four structural proteins–nucleocapsid (N), spike (S), envelope (E), and membrane (M) proteins. The S protein of the virus mediates viral entry into host cells [7]. Despite the proofreading activity [8], SARS-CoV-2 genomic RNA had undergone frequent mutations as the COVID-19 pandemic drags on, leading to various variants. The D614G mutation, N501Y mutation, E484K mutation, and the other high-frequency mutations had shown the ability to affect the virus’s properties [9–12]. Alpha, Beta, Gamma, Delta, and Omicron were five SARS-CoV-2 variants designated as Variants of Concern (VOC) by WHO. Omicron variant was first reported to WHO on November 24, 2021, from South Africa, while the first known laboratory-confirmed case was identified from a specimen collected on November 9, 2021. Compared to other variants, Omicron had shown an increased ability to spread within the community [13]. It had overtaken the Delta variant and become the dominant strain circulating all over the world.

After the identification of SARS-CoV-2, it was discovered that this virus used primarily the human angiotensin-converting enzyme 2 (hACE2) to gain entry into host cells, but was incapable of using the murine ortholog mACE2 as a receptor [14, 15]. However, subsequent studies indicated that laboratory mice can be effectively infected not only by the mouse-adapted SARS-CoV-2 strains [16–20], but also by different SARS-CoV-2 VOC, including Alpha, Beta, Gamma, Omicron [21–25], which positions rodents as a potential intermediate host for SARS-CoV-2 [21, 25, 26] that promotes the zoonotic transmission. The spillover infection of mice was attributed to the mutations in the receptor-binding domain (RBD) of S glycoprotein. Among the RBD mutations, the N501Y mutation was identified as the key point mediating the cross-species process to mice by endowing mACE2 binding [10], which could be enhanced by other neighboring mutations within RBD, such as Q493H and K417N [17, 27]. However, it is unclear whether mutations outside of the RBD may regulate mouse infectivity by N501Y mutation, and how this regulation may work out if it does.

In this study, we managed to explore this question by taking advantage of MA-SARS2, a mouse-adapted SARS-CoV-2 strain [20]. The spike protein of MA-SARS2 contains N501Y mutation and two extra-RBD mutations: Ins215KLRS for insertion of KLRS at the position of 215th amino acid in the NTD region and H655Y mutations in the SD region linking S1 to S2 [20]. We demonstrated that either Ins215KLRS or H655Y could, respectively, enhance N501Y-endowed mouse infection via mACE2, which was further potentiated by a combination of Ins215KLRS and H655Y. Mechanistically, this long-range regulation may work out by a local conformation change around the furin-cleavage site by H655Y, and an altered intramolecular interaction by Ins215KLRS leading to a more flexible RBD that facilitates receptor binding. Moreover, this mechanism may underly the high mouse-infectiousness of the Omicron variant, whose Spike protein contains Ins214EPE and H655Y mutations. Collectively, our study sheds light on the cooperation between distant Spike mutations in promoting virus infectivity.

## Results

### MA-SARS2 spike confers mouse infectivity

**Fig. 1:**
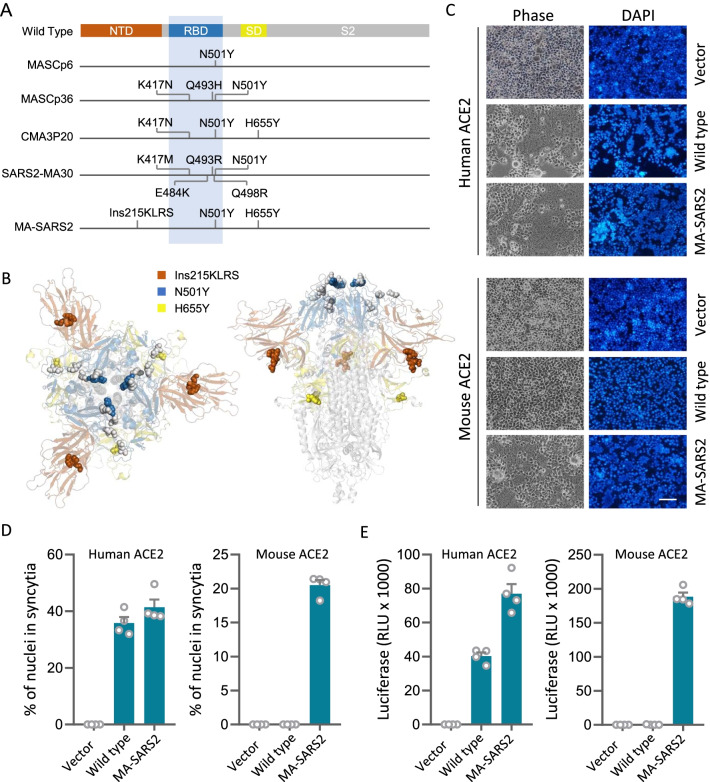
MA-SARS2 spike grants mouse infectivity **A** Schematic illustration of mutations in the spike protein of mouse-adapted strains containing N501Y. NTD (brown): N terminus domain; RBD (blue): receptor-binding domain; SD (yellow): subdomain; S2 (gray): subunit 2. **B** Top (left) and side (right) view of S glycoprotein of mouse-adapted strains. Spheres represent mutations, brown: Ins215KLRS, blue: N501Y, yellow: H655Y, gray: other mutations in RBD. **C** Representative images of syncytia formation upon expression of the indicated S glycoprotein in 293T cells expressing human ACE2 (hACE2) and mouse ACE2 (mACE2). Bar: 100 μm. **D** Quantification of syncytia formation. Data are the mean ± SD of results from 4 fields (10× objective lens). More than three replicates were performed. **E** Expression of the luciferase reporter in 293T-hACE2 and 293T-mACE2 cells upon infection of viruses pseudotyped with Wild type or MA-SARS2 S glycoproteins. Data are the mean ± SD of triplicate measurements

To explore the potential cooperation between N501Y and other mutations on spike protein, we compared the mutations of the spike from five mouse-adapted strains that all carry N501Y mutation [16–20]. As shown in Fig. 1 A, there are four additional mutations, other than N501Y, identified within the RBD region, including K417, E484, Q493, and Q498. All these mutations are positioned within a spatially narrow cluster that conceivably impacts spike-ACE2 interaction (Fig. 1B), which is in agreement with previous studies [28]. Interestingly, the spike of MA-SARS2 mouse-adapted strain contains two extra-mutations, Ins215KLRS and H655Y [20], that are not only outside of the RBD region in sequence (Fig. 1A), but also distant to the receptor-binding domain in spatial structure (Fig. 1B). Therefore, the MA-SARS2 spike was selected for further investigation. We first examined the membrane fusion ability of the MA-SARS2 spike by employing the syncytia formation assay, where the MA-SARS2 spike was co-expressed with hACE2 or mACE2 to allow spike-ACE2 interaction and fusion of neighboring cells to form syncytia as described before [6, 29]. As a result, the MA-SARS2 spike efficiently induced syncytia in hACE2-expressing cells with an efficiency comparable to that of the wild type spike protein (Fig. 1C, D), and exhibited a high specificity in inducing syncytia in mACE2-expressing cells while the wild type spike did not at all (Fig. 1C, D). Moreover, the viruses pseudotyped with the MA-SARS2 spike, but not the wild type spike, efficiently infected mACE2-expressing cells (Fig. 1E), which is consistent with an essential role of N501Y in dictating mouse tropism [10, 30]. Collectively, these results are compatible well with that MA-SARS2 is capable of utilizing mouse ACE2 to achieve host entry.

### Ins215KLRS and H655Y assist N501Y to enhance receptor binding and mouse infectivity

**Fig. 2:**
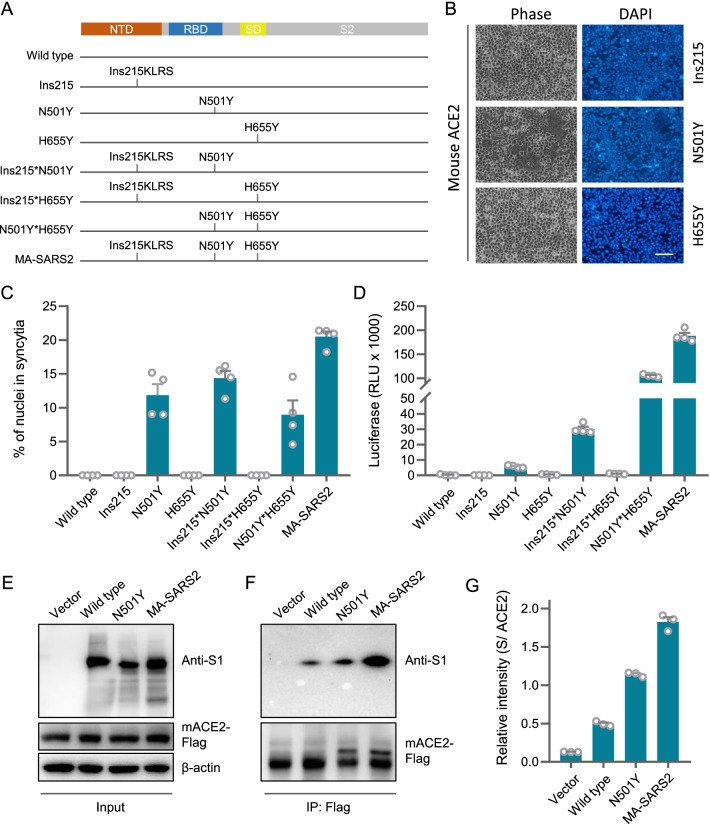
Ins215KLRS and H655Y enhance receptor binding and mouse infectivity by N501Y. **A** Schematic illustration of different mutants. **B** Representative images of syncytia formation upon expression of the indicated S glycoprotein in 293T-mACE2. Bar: 100 μm. **C** Quantification of syncytia formation upon expression of the indicated S glycoprotein in 293T-mACE2. Data are the mean ± SD of results from 4 fields (10× objective lens). **D** Expression of the luciferase reporter in 293T-mACE2 cells upon infection of viruses pseudotyped with indicated S glycoproteins. Data are the mean ± SD of triplicate measurements. **E**,** F** Immunoblot of the total cell lysate and immunoprecipitates (IP) derived from the 3*Flag tagged-mACE2 expressing cells transfected with SARS2-S WT or mutant plasmids. **G** quantification of S/ ACE2 was performed Data are the mean ± SD of triplicate measurements

To explore whether Ins215KLRS and H655Y, the two mutations outside of the RBD region, may influence N501Y-endowed mouse infectivity, we first compared the MA-SARS2 spike with the N501Y spike in their abilities to induce syncytia and mediate infection. For this sake, a set of constructs were made as indicated in Fig. 2A. Interestingly, while Ins215KLRS and H655Y mutations, respectively on their own, were incapable of inducing syncytia in mACE2-expressing cells (Fig. 2B, C), they seemed to cooperate with N501Y to enhance the fusogenic ability of spike protein as their absence significantly compromised syncytia formation in cells expressing mACE2 (Fig. 2B, C). And this cooperation was also indicated in their ability to promote the infection of mACE2-expressing cells by the corresponding pseudoviruses (Fig. 2D). Consistently, the presence of Ins215KLRS and H655Y mutations increased the receptor binding of spike with N501Y mutation to mACE2 as determined by an immunoprecipitation assay (Fig. 2E, F and G). Collectively, these results demonstrated that Ins215KLRS and H655Y could assist N501Y to improve the infectivity of the virus to mACE2-expressing cells.

### Ins215KLRS and H655Y promote a flexible spike conformation

**Fig. 3:**
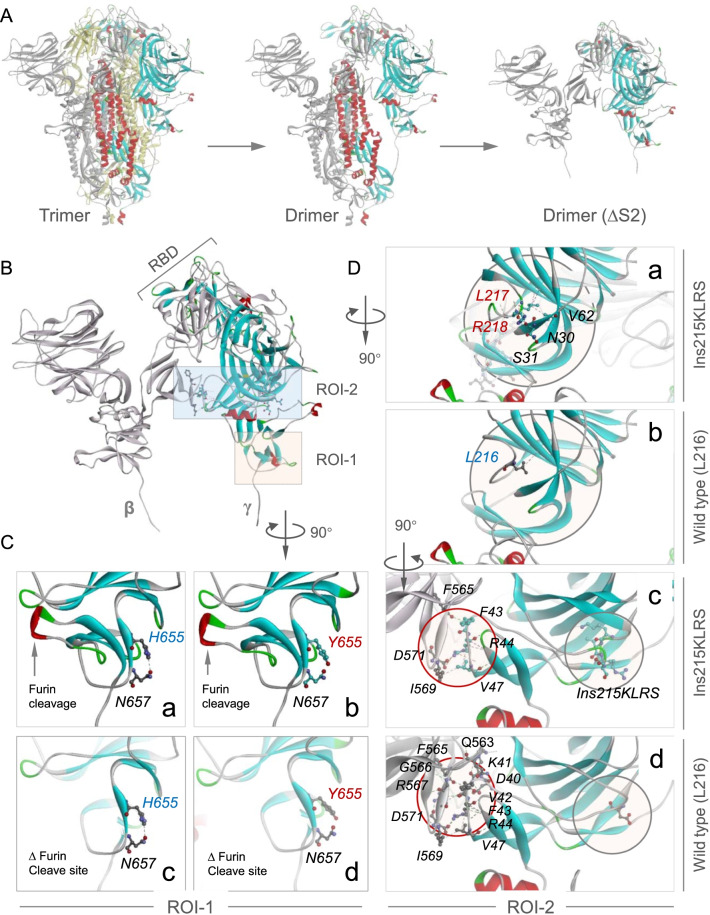
Three-dimensional structure modeling of the MA-SARS2 spike. **A** Side view of S glycoprotein whose chain colored in Yellow/ Gray/ Rainbow, Drimer (ΔS2): two protomers lacking S2 subunit. **B** Zoomed-in of Drimer (ΔS2) as indicated. ROI-1: region 1 of interest, ROI-2: region 2 of interest; β, γ: S1 subunit of S glycoprotein. **C** Zoomed-in of ROI-1 after clockwise rotation by 90 degrees. Images show the interactions between H655 (a, c) or Y655 (b, d) with N657 in the presence or absence of furin cleavage finger. **D** Zoomed images of ROI-2 after clockwise flipping by 90 degrees (a, b) and subsequent clockwise rotation by 90 degrees (c, d). Images a, b show the interactions between two chains, c, d show the interactions between wild type (L216) or Ins215KLRS and other amino acids. Gray circle: Ins215KLRS mutation

To illustrate the structural mechanism whereby Ins215KLRS and H655Y enhance N501Y-endowed mouse infectivity, we constructed a 3-dimension structure for MA-SARS2 spike trimer by SWISS-MODEL modeling with 7df4.pdb as the template [31], which was sequentially destructured to produce a sub-dimer between β and γ protomers truncated in S2 domain (∆S2) (Fig. 3A). The structural analysis identified two regions of interest (ROI) defined by H665Y (ROI-1) and Ins215KLRS (ROI-2), respectively (Fig. 3B). Given the spatial distance to RBD that contains N501Y mutation, it’s unlikely that H655Y and Ins215KLRS might influence receptor-binding of N501Y-RBD to mACE2 in a direct way. Instead, we speculated a mutation-induced local conformation change may impose an indirect impact in a long-range. In line with this idea, we find that the H655Y mutation within ROI-1 disrupted the interaction between H655 and N657, leading to a looser loop wrapping the furin cleavage finger that protruding through the loop, which conceivably facilitates cleavage-dependent shielding of S1 to promote host entry. Meanwhile, the insertion of KLRS after L215 resulted in increased local interaction with N30 and S31 on a neighboring chain (gray circles, Fig. 3B, D-a and b), which extends to an inter-protomer interacting face (red circles) that links directly to the RBD of neighboring protomer (Fig. 3D-c, d). Importantly, corresponding to the increased local interaction, there is a dramatically reduced inter-protomer interaction (from 6 to 3 interaction pairs as indicated in Fig. 3D-c, d), which is expected to promote receptor-binding-induced RBD shielding, thus unsheathing the fusogenic S2 to mediate host entry. Together, the above data are consistent with the notion that Ins215KLRS and H655Y induced a flexible spike conformation to promote N501Y-endowed mouse infectivity.

### Ins214EPE and H655Y are required for the mouse infectivity of the Omicron variant

**Fig. 4:**
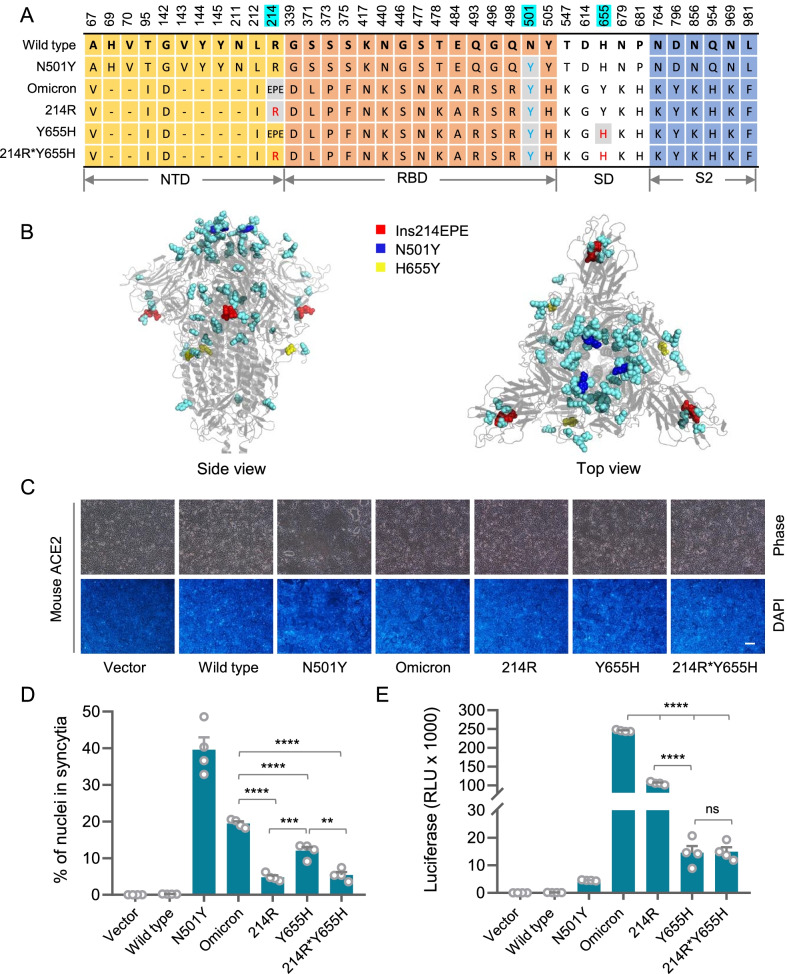
Ins214EPE and H655Y are essential for the infectivity of Omicron to mice.** A** Several reverse-mutants for H655Y and Ins214EPE by mutating them back to the corresponding wild-type amino acids while keeping the N501Y mutation based on the Spike protein of the Omicron variant. NTD: N terminus domain; RBD: receptor-binding domain; SD: subdomain; S2: subunit 2. Cyan: three mutant sites. **B** Side and top view of S glycoprotein of Omicron. Blue: N501Y. Red: Ins214EPE. Yellow: H655Y. Cyan: other spike mutations of Omicron. **C**,** D** Representative images (C) and quantification (D) of syncytia formation upon expression of the indicated S glycoprotein in 293T-mACE2. Data are the mean ± SD of results from 4 fields (10× objective lens). More than three replicates were performed. **E** Expression of the luciferase reporter in 293T-mACE2 cells upon infection of viruses pseudotyped with Wild type or mutant type of S glycoproteins as indicated. Data are the mean ± SD of triplicate measurements

The latest studies identified Omicron infection in rodents including mice and hamsters [21, 25, 26], and transmitting back to humans leading to an onward human-to-human transmission [26], supporting the zoonotic transmission of the emerging SARS-CoV-2 variant. The Omicron variant is known to have over 60 mutations, of which 34 mutations are on the spike protein [32]. Interestingly, in addition to the N501Y mutation in the RBD region that confers mouse infectivity [10, 30], the Omicron spike also harbors two non-RBD mutations: H655Y and Ins214EPE, an insertion within the ROI-2 intimately close to the L215 (Fig. 4A, B) identified in Fig. 4D, suggesting that these two mutations might play a role in enhancing mouse infectivity. To test this idea, we made several reverse-mutants for H655Y and Ins214EPE by mutating them back to the corresponding wild-type amino acids while keeping the N501Y mutation (Fig. 4A). The cell fusion assay showed that reverse-mutagenesis of Ins214EPE and H655Y, either individually or combinedly on the Omicron spike, dramatically compromised syncytium formation in mACE2-expressing cells (Fig. 4C, D). In agreement, the mouse infectivity of Omicron spike-pseudotyped viruses was also significantly inhibited upon reverse-mutation of Ins214EPE and H655Y (Fig. 4E). These results suggest that the mutations of Ins214EPE and H655Y are critical for mouse infection of the Omicron variant, which might employ a similar strategy whereby the mouse-adapted MA-SARS2 strain utilized as identified above to enhance mouse infectivity. Of note, the reverse-mutation of Ins214EPE and H655Y clearly imposed different effects, in terms of the extent, on syncytium formation and pseudovirus infection (Fig. 4D, E), where the 214R mutation primarily inhibited syncytium formation and the Y655H mutation played a dominant role in pseudovirus infection. This may be related to different routes by which Ins214EPE and H655Y promote host entry of SARS-CoV-2 as discussed below.

## Discussion

Host entry of SARS-CoV-2 is initiated by the binding of Spike protein to its receptors on the cell surface [33, 34], where the RBD of Spike protein engaged with ACE2 to mediate two major routes to make infection. The first route takes place at the plasma membrane, where the membrane proteases, such as TMPRSS2, could make a second cleavage on Spike protein that has been processed by the furin protease during virus production. By this route, direct membrane fusion occurs on the plasma membrane, leading to the release of viral genetic materials into the victim cell, therefore it was termed the plasma membrane route. The second route works out by internalizing virus into endosomes, where the Spike protein could be cleaved by the endosomal cathepsins to allow membrane fusion and release of viral genetic materials, therefore it was termed as the endosomal route. Alternative utilization of these two routes was believed to confer SARS-CoV-2 high infectiousness [35].

In our study, the Ins214EPE mutation is required for efficient membrane fusion to an extent more prominent than that of the H655Y mutation (Fig. 4D), indicating that Ins214EPE may preferentially promote Omicron infection via the plasma route. This is in good agreement with its structural role in promoting unsheathe of the fusogenic S2 by producing a more flexible Spike complex (Fig. 3D). Conversely, despite a relatively mild effect on syncytium formation (Fig. 4D), H655Y mutation profoundly impacts virus infection (Fig. 4E), suggesting that H655Y mutation is critical for Omicron infection which is unlikely to go through the plasma route, but instead through the endosomal route. In line with this notion, the H655Y mutation takes place on the loop wrapping the furin cleavage finger, leading to a conformation change that conceivably impacts the processing of Spike protein by furin protease, a step that turned out to be essential for Spike-induced membrane fusion [6]. This result suggests that the H655Y-carrying Omicron may preferentially infect target cells via the endosomal route, which is consistent with the latest study by Yamamoto et al., who reported in bioRxiv that the H655Y mutation is responsible for enhanced endosomal entry and reduced cell surface entry of Omicron variant [36].

Intriguingly, the compromised membrane fusion by H655Y mutation may also set a basis for the less symptomatic clinical manifestation of Omicron patients. It was reported that cell fusion played important role in the pathogenesis of COVID-19 [37]. On one hand, SARS-CoV-2 infection could induce syncytia formation in a way dependent on the presence of a unique bi-arginine motif around the furin cleavage site that dictates cell-cell fusion [6]. The syncytia resulting from infection-induced cell-cell fusion may actively internalize lymphocytes to form heterotypic cell-in-cell structures [6, 38], a pathological phenotype mostly documented in human tumors [39–44], and genetically controlled by a set of core elements, including adherens junctions, actomyosin and mechanical ring [45–49]. The formation of cell-in-cell structure frequently leads to the death of the internalized lymphocytes in an acidified lysosome [6, 50–52], contributing to lymphopenia which was believed to be a causal factor linked to severe COVID-19 [53–56]. On the other hand, the multi-nucleated syncytia tended to produce cytoplasmic chromatin, leading to the formation of naked micronuclei/DNA, which could readily active the DNA damage response, and cGAS-STING signaling and subsequently anti-viral innate immunity [3, 57]; meanwhile, the formation of syncytia may lead to the activation of necrotic cell death by pyroptosis [58], thus promoting inflammation causing local tissue damages. Whereas, the Omicron variant exhibits impaired ability to induce cell-cell fusion by the H655Y mutation, and is therefore relatively less pathogenic as compared with other variants of concern.

Despite alternative receptors that have been reported for SARS-CoV-2, ACE2 is the major cellular receptor that mediates host entry through binding to the RBD region of Spike protein [59]. Therefore, mutations in the RBD region were extensively investigated for their impacts on the infectivity of different SARS-CoV-2 variants. Previous studies, largely based on the mouse-adapted strains, identified N501Y, K417, E484, Q493 and Q498 as the functional mutations that regulate mouse infectivity [16–20], among which, the N501Y mutation was identified as the key mutation that mediates cross-species infection to mice [10] with the assistance of other RBD mutations [30]. Of note, in addition to the RBD region, mutations were also identified in non-RBD regions of Spike protein for some mouse-adapted strains with unclear functional implications [20, 30].

## Conclusions

In this study, we took advantage of the MA-SARS2 mouse-adapted strain [20] to explore the long-range regulation of viral infectivity by mutations outside of the RBD region. Structural modeling showed that the two non-RBD mutations of Ins215KLRS and H655Y are spatially positioned away from the RBD region as expected. Nevertheless, they profoundly regulated the mouse infectivity dictated by N501Y mutation as evidenced by significantly altered syncytium formation and pseudovirus infection. This is related to conformation changes that potentially impact Spike processing by furin protease by H655Y mutation in the SD region and reduce inter-protomer interactions to promote S1 shielding/S2 unsheathing by Ins215KLRS in the NTD region (Fig. 3). Thus, our study provides a proof-of-concept example for long-range regulation of RBD-mediated infectivity by non-RBD mutations, which would help elucidate functional interactions of RBD residues with other non-RBD mutations. Meanwhile, our study provides genetic evidence supporting the zoonotic infection and transmission in rodents including mice and hamsters, which should be taken into account during the prevention of emerging SARS-CoV-2 variants such as Omicron.

## Methods

### Bioinformatics

The 3D structure modeling of SARS-CoV-2 S glycoprotein containing Ins215KLRS, N501Y, H655Y was performed by the Modelling algorithm at SWISS-MODEL (https://swissmodel.expasy.org/) with the template of 7df4.pdb reported by Xu et al. [60] from RSCB protein data bank (http://www.rcsb.org/). The template of 7tnw.pdb reported by Mannar et al. [61] was used for the modeling of Omicron spike protein.

### Cell culture

The 293T-hACE2 and 293T-mACE2 cells were maintained in DMEM (MACGENE Tech Ltd., Beijing, China) supplemented with 10% fetal bovine serum (Kang Yuan Biol, Tianjin, China) and 1% Penicillin-Streptomycin (MACGENE Tech Ltd., Beijing, China). All cells were incubated with 5% CO_2_ at 37 °C. The 293T-hACE2 and 293T-mACE2 were stable cell lines expressing hACE2 and mACE2, respectively (Additional file 1: Table S1).

### Constructs

The codon-optimized SARS-CoV-2 S cDNA was synthesized at Genscript Biotech Corporation (Nanjing, China). The wild type or the mutant S genes of SARS-CoV-2 were cloned into pSecTag2-Hygro-A through seamless homologous recombination. Please find in supplementary tables for detail information on the constructs and primers used in this study (Additional file 1: Table S2 and Table S3).

### Cell fusion

For cell fusion assay, about 6 × 10^5^ cells were plated per well in 6-well plate precoated with type I collagen (354236, BD Biosciences) and cultured for 24 h. Cells were then transfected with respective constructs by Lipofectamine LTX and Plus Reagent (Invitrogen, 1784283, USA) following the protocol provided. Images of 4 fields (10× objective lens) were taken on Hoechst-stained cells 36 h post transfection by Nikon microscope. Syncytia area was performed by NIS elements AR software (Nikon, Japan).

### Pseudovirus production

The mouse sarcoma virus (MSV)-based SARS-CoV-2 S, and SARS-CoV-2 mutants pseudotypes were prepared as previously described [9]. HEK293T cells were co-transfected with an S encoding-plasmid, a Gag-Pol packaging construct (Addgene, 8449, USA; Additional file 1: Table S4) and the pQCXIP retroviral vector (Clontech, USA) expressing a luciferase reporter by using Lipofectamine LTX and Plus Reagent (Invitrogen, 1,784,283, USA) according to the manufacturer’s instructions. Cells were incubated for 6 h at 37 °C with transfection medium. Then transfection medium was changed with DMEM containing 10% Fetal Bovine Serum (FBS) was added for 48 h. The supernatants were then harvested and filtered through 0.45 μm membranes and then frozen at −80 ℃.

### Pseudovirus assay

293T-hACE2 cells and 293T-mACE2 cells were plated into 96 well plates at a density of 0.5 × 10^4^ per well for 16 h. About 1.15 × 10^4^ copies of virus in the volume of 50 µL and 50 µL DMEM was added to the wells. After 12 h, 100 µL 10% FBS and 1% PenStrep containing DMEM was added to the cells. Following the 48 h-infection, 100 µL One-Glo-EX (Promega, E6120) was added to the cells in equivalent culturing volume and incubated in the dark for 10 min prior to reading on an Enspire 2300 multilable reader (Perkin Elmer, USA). Measurements were done at least in triplicate and relative luciferase units (RLU) were plotted.

### Immunoprecipitation

Immunoprecipitation (IP) was performed to determine the affinities between S and ACE2 as described before [62]. In brief, about 1 × 10^6^ 293T-hACE2 or 293T-mACE2 cells were plated per well in 6-well plates and cultured for 16 h at 37 °C before transfected with different plasmid. After 48 h, cells were lysed by the ice-cold IP lysis buffer (20 mM Tris, 0.1 M NaCl, 0.1% NP40, 5 mM EDTA in ddH_2_O and pH = 8) with phosphatase inhibitor cocktail (CWBiotech, Beijing) and protease inhibitor cocktail (CWBiotech, Beijing), and IP experiment was performed using the protein A/G agarose (Beyotime Biotechnology). Then, lysates were further cracked with ultrasound (power 40%, work 6 s, stop 9 s, 5 times in total). After being centrifuged at 12,000 rpm for 10 min, the supernatant was collected, and a small amount of which was for input. The remaining supernatant was blocked with 20 ml protein A/G beads (pre-washed with cold IP lysis buffer) for 1 h. Flag-Tag (Abbkine) or anti-IgG was incubated with protein lysate removed protein A/G agarose at 4 °C overnight. The next day, add 30 ml protein A/G beads into the protein lysate and continue to incubate for 2 h, and beads were washed extensively with cold IP lysis buffer. IP products were harvested using denaturing elution and subjected to Western blot analysis to detect protein-protein interactions (Additional file 1: Table S5).

### Statistics

Data were expressed as means with standard deviations (SD). *P*-values were calculated using two-tailed Student’s t-test from GraphPad Prism software, and *P*-values less than 0.05 were considered statistically significant.

## Supplementary Information

**Additional file 1: Table S1.** Cells used in this study. **Table S2.** Information for constructs made in this study. **Table S3.** Primers used in this study. **Table S4.** Constructs from Addgene. **Table S5.** Antibodies used in this study.

## Data Availability

All data generated or analyzed during this study are included in this published article [and its supplementary information files].

## Abbreviations

SARS-CoV-2: Severe acute respiratory syndrome coronavirus 2
COVID-19: Coronavirus disease 2019
ACE2: Angiotensin converting enzyme 2
RBD: Receptor-binding domain
NTD: N-terminal domain
SD: Subdomain
VOC: Variants of concern
WHO: World health organization
hACE2: Human angiotensin converting enzyme 2
mACE2: Mouse angiotensin converting enzyme 2
