# Supplementary material for: Long-range enhancement of N501Y-endowed mouse infectivity of SARS-CoV-2 by the non-RBD mutations of Ins215KLRS and H655Y
Source: Biol Direct. 2022 Jun 5;17:14. doi: 10.1186/s13062-022-00325-x (PMC9167559; doi:10.1186/s13062-022-00325-x)
Supplement: Supplementary file 1 — Additional file 1: Table S1. Cells used in this study. Table S2. Information for constructs made in this study. Table S3. Primers used in this study. Table S4. Constructs from Addgene. Table S5. Antibodies used in this study. [file 13062_2022_325_MOESM1_ESM.docx]

**Supplemental Tables**

Table S1. Cells used in this study.

| Cell lines | Source | Tissue Source | Cell type | Growth Properties | Culture medium |
| --- | --- | --- | --- | --- | --- |
| HEK-293T-hACE2 | constructed in the lab | human, kidney | epithelial cell | adherent | DMEM+10% FBS+1% Penicillin-Streptomycin |
| HEK-293T-mACE2 | constructed in the lab | human, kidney | epithelial cell | adherent | DMEM+10% FBS+1% Penicillin-Streptomycin |

Table S2. Information for constructs made in this study.

| Plasmids | Construct Method | Backbone | Site | DNA | Primer | Primer Sequence (5'→3') |
| --- | --- | --- | --- | --- | --- | --- |
| pSecTag2-CoV2-S | Homologous Recombination | pSecTag2 Hygro A | *Xho* I | CoV2-S | SARI-F1 | AGCTTGGTACCGAGCTCGCAGTGCGTCAATCTGACAACTCG |
|  |  |  |  |  |  |  |
|  |  |  | *Bam*H I |  | SARI-R1 | TTCGGGCCCTCCTCGAGCGGTGTAATGCAGCTTCACGC |
|  |  |  |  |  |  |  |
| pSecTag2-CoV2-N501Y | Homologous Recombination | pSecTag2 Hygro A | *Xho* I | CoV2-N501Y-1 | HA-S1-F | ACGAAGCTTGGTACCGAGCTCG |
|  |  |  |  |  | N501Y-R | CCCACGCCGTATGTGGGCTGGAAGCCGTAGGAC |
|  |  |  | *Bam*H I | CoV2-N501Y-2 | N501Y-F | CAGCCCACATACGGCGTGGGCTATCAGCCTTAC |
|  |  |  |  |  | S2-R | TGAGTTTTTGTTCGGGCCCTCCTC |
| pQCXIP-EGFP-Luciferase | Cohesive End Ligation | PNL4.3.luc.R-E | *Eco*R V | Luciferase | Luc-F1 | ACGGTACCGCGGGCCACCATGGGTGGCGCGGCCGC |
|  |  |  |  |  |  |  |
|  |  |  | *Bam*H I |  | Luc-R1 | AGAGCCTGGACCACTGATCTAGGTCTCGAGCAATTTGGACTTTCCGCCC |
|  |  |  |  |  |  |  |
| pQCXIP-hACE2 | Cohesive End Ligation | pQCXIP-N1 | *Sbf* I | human ACE2 | ACE2-F1 | CATTGGAACGGACCTGCAGCCACCATGTCAAGCTCTTCC |
|  |  |  | *Not* I |  | ACE2-R1 | ATTATGATCTAGAGTCGCTCACTTGTCATCGTCATCCTTGTAGTCG |
| pQCXIP-mACE2 | Cohesive End Ligation | pQCXIP-N1 | *Xho* I | mouse ACE2 | Mouse-ACE2-F1 | CGGCCGCACCGGGATCTCGAATGTCCAGCTCCTCCTGGCTC |
|  |  |  | *Pac* I |  | Mouse-ACE2-R1 | CATGGTCTTTGTAGTCACGCGTCACCGGAAAGGAAGTCTGAGCATCATC |

Table S3. Primers used in this study.

| Primer | Primer Sequence (5'→3') |
| --- | --- |
| A67V-DEL69 70-R | CAGGAAGGGATCATTACAAAACTGGAAC |
| A67V-DEL69 70-F | GTGACCTGGTTCCACGTGATCAGCGGCACCAATGGCAC |
| T95I-F | CGAGAAGAGCAACATCATCAG |
| T95I-R | TGATGTTGCTCTTCTCGATAGAGGCGAAGTACACGC |
| G142D/DEL143-145-F | GTTTTGTAATGATCCCTTCCTGGACCACAAGAACAATAAGAGCTGGATG |
| G142D/DEL143-145-R | CAGGAAGGGATCATTACAAAACTGGAAC |
| Del211-L212I-INS214EPE-F | CTAAGCACACCCCCATCATCGTGCGCgagcccgagGACCTGCCTCAGGGCTTCAGCG |
| Del211-L212I-INS214EPE-R | GATGGGGGTGTGCTTAGAGTAG |
| G339D-R | AAATGGGCACAGGTTTGTGATATTAGG |
| G339D-F | CTAATATCACAAACCTGTGCCCATTTGACGAGGTGTTCAACGCAACCCG |
| S371L-S373P-S375F-R | GTTGTACAGCACGCTATAGTCGGC |
| S371L-S373P-S375F-F | GACTATAGCGTGCTGTACAACCTGGCCCCCTTCTTCACCTTTAAGTGCTATGGCG |
| K417N-F | CATCGCAGACTACAATTATAAGCTGCC |
| K417N-R | TAATTGTAGTCTGCGATGTTGCCTGTCTGGCCGGG |
| N440K-G446S-R | GTTGCTGTTCCAGGCGATCAC |
| N440K-G446S-F | GTGATCGCCTGGAACAGCAACAAGCTGGATTCCAAAGTGAGCGG |
| S477N-T478K-F | CAGAAATCTACCAGGCCGGCAACAAGCCTTGCAATGGCG |
| S477N-T478K-R | CAGAAATCTACCAGGCCGGCAACACCCCTTGCAATGGCGTG |
| E484A-R | GTGGGAAATAACAGTTAAAGCCGGCCACGCCATTGCAAGG |
| E484A-F | GCCGGCTTTAACTGTTATTTCCCACTC |
| Q493R-G496S-Q498R-R | GAGTGGGAAATAACAGTTAAAGCCC |
| Q493R-G496S-Q498R-F | GGCTTTAACTGTTATTTCCCACTCAGATCCTACAGCTTCAGGCCCACATACGGC |
| N501Y-Y505H-F | CCCACATACGGCGTGGGCCACCAGCCTTACCGCG |
| N501Y-Y505H-R | GATAGCCCACGCCGTATGTGGG |
| T547K-R | CAGGCCGTTGAAGTTGAAGTTCAC |
| T547K-F | GAACTTCAACTTCAACGGCCTGAAGGGCACAGGCGTGCTGACC |
| N679K-P681H-R | TGTCTGGGTCTGGTAAGAGGC |
| N679K-P681H-F | CTCTTACCAGACCCAGACAAAGTCTCACAGAAGAGC |
| N764K-R | CAGCTGGGTACAAAAGCTGCC |
| N764K-F | GGCAGCTTTTGTACCCAGCTGAAGAGAGCCCTGACAGGCATCGC |
| D796Y-R | CTTGATAGGGGGGGTCTTGTAG |
| D796Y-F | CTACAAGACCCCCCCTATCAAGTACTTTGGCGGCTTCAATTTTTCC |
| N856K-R | AAACTTCTGGGCGCAGATCAG |
| N856K-F | CTGATCTGCGCCCAGAAGTTTAAGGGCCTGACCGTGCTGC |
| Q954H-R | ATTCACCACATCCTGGAGCTTGC |
| Q954H-F | CAAGCTCCAGGATGTGGTGAATCACAACGCCCAGGCCCTG |
| N969K-R | GCTGCTCAGCTGCTTCACCAG |
| N969K-F | CTGGTGAAGCAGCTGAGCAGCAAGTTCGGCGCCATCTCTAGCG |
| L981F-R | GATGTCATTCAGCACGCTAGAGATG |
| L981F-F | CATCTCTAGCGTGCTGAATGACATCTTCAGCCGGCTGGACAAGG |
| Ins214EPE R-R | GCGCACGATGATGGGGGTGTGC |
| Ins214EPE R-F | CCCCATCATCGTGCGCGACCTGCCTCAGGGCTTCAGC |
| Y655H-R | CTCATAGGAATTGTTCACGTGCTCTGCTCCGATCAGGCATCC |
| Y655H-F | ACGTGAACAATTCCTATGAGTGCG |
| Ins215KLRS-F | TAAGCTGAGAAGCCTGCCTCAGGGCTTCAG |
| Ins215KLRS-R | GCAGGCTTCTCAGCTTATCGCGCACCAGGTTGATGG |
| H655Y-F | ACGTGAACAATTCCTATGAGTGCG |
| H655Y-R | ATAGGAATTGTTCACGTACTCTGCTCCGATCAGGCATCC |

Table S4. Constructs from Addgene.

| Name | Source | Cat. No | Inserts | Purpose |
| --- | --- | --- | --- | --- |
| pCMV-VSV-G | Addgene | 8454 | VSV-G | Envelope protein for producing lentiviral and MuLV retroviral particles |
| pUMVC | Addgene | 8449 | gag-pol | Packaging plasmid for producing MuLV retroviral particles. |

Table S5. Antibodies used in this study.

| Antibody | Company | Cat. No | Source | Type | Dilution |
| --- | --- | --- | --- | --- | --- |
| β-Actin | Poteintech | 60008-1-lg | Mouse | Monoclonal | WB 1:2000 |
| ACE2 | Proteintech | 21115-1-AP | Rabbit | Polyclonal | WB 1:1000 |
| Spike S1 | ABclonal | A20136 | Rabbit | Polyclonal | WB 1:2000 |
| Flag | Abbkine | A02010 | Mouse | Monoclonal | IP: 1:400 |
| Anti-rabbit IgG HRP | CST | 7074 | Goat |  | WB 1:3000 |
| Anti-mouse IgG HRP | CST | 7076 | Horse |  | WB 1:3000 |
